# The Role of Comprehensive Brain Perfusion and Whole-Body CT Using Split-Bolus Injection in Diagnosing Multiple Thromboembolism in a Wake-Up Stroke Patient: A Case Report

**DOI:** 10.7759/cureus.86726

**Published:** 2025-06-25

**Authors:** Takayuki Inomata, Koji Nakaya, Takaya Sasaki, Hiroto Shiozaki, Yasuto Noda

**Affiliations:** 1 Department of Radiological Technology, Faculty of Health Science, Suzuka University of Medical Science, Suzuka, JPN; 2 Department of Radiological Technology, Fuji City General Hospital, Fuji, JPN; 3 Department of Neurosurgery, Fuji City General Hospital, Fuji, JPN

**Keywords:** brain perfusion ct, cardio-cerebral infarction, left atrial thrombus, multiple thromboembolisms, split-bolus contrast injection, wake-up stroke, whole-body ct

## Abstract

Brain computed tomography (CT) perfusion imaging is essential for determining patient eligibility for mechanical thrombectomy in wake-up stroke cases. CT scanning is also valuable for enabling simultaneous whole-body imaging, which supports access route evaluation and helps exclude differential diagnoses such as aortic dissection. In this report, we describe comprehensive brain perfusion CT and whole-body imaging for diagnostic evaluation in a 91-year-old female patient who presented to the emergency department with a suspected wake-up stroke. The use of the split-bolus contrast injection method, combined with a low tube voltage, enabled a simplified protocol that used a minimal contrast medium dose, achieving CT attenuation values of ≥400 Hounsfield units throughout the major thoracoabdominal vessels. This enhanced vascular visualization supported a prompt and accurate diagnosis. We identified an exceptionally rare case of multiple simultaneous thromboembolisms, including cerebral infarction due to brachiocephalic artery occlusion, pulmonary thromboembolism, left atrial thrombus, and myocardial infarction. All diagnoses were made in a single imaging session. Although rare, concurrent aortic dissection and myocardial infarction have been reported in patients with acute ischemic stroke, underscoring the clinical importance of comprehensive diagnostic evaluation. This case illustrates the utility of integrated brain perfusion and whole-body CT scanning in identifying multi-site thromboembolisms.

## Introduction

Acute ischemic stroke (AIS) outcomes have markedly improved with the introduction of intravenous recombinant tissue plasminogen activators and mechanical thrombectomy (MT) [1]. Computed tomography (CT) and magnetic resonance imaging are essential for diagnosing AIS. In particular, for patients with a suspected stroke of unknown onset time, such as a wake-up stroke, whole-brain CT perfusion (CTP) imaging plays a critical role in determining eligibility for MT based on assessments of the ischemic core and penumbra [2]. Additionally, CT enables a series of whole-body scans to be performed following a brain scan, allowing confirmation of vascular access routes. This supports the selection of appropriate vessels and devices for MT and has been shown to help reduce the number of MT procedures [3]. Moreover, some patients presenting with suspected AIS may have an underlying acute aortic dissection [4]. Although the incidence is reported to be as low as 0.31%, this rare condition must have substantial implications for prognosis and treatment decisions. Therefore, combining brain CTP with whole-body CT scanning enables comprehensive diagnosis and facilitates treatment planning, including determination of MT eligibility in suspected AIS cases.

One concern with CT imaging is the potential effect of contrast medium (CM) on renal function. However, recent studies suggest that intravenous CM poses a limited risk of nephrotoxicity [5-7]. Furthermore, the use of low tube voltages can reduce the volume of CM required while maintaining adequate contrast enhancement, thereby minimizing renal burden [8]. The split-bolus contrast injection method (SPBI) is a technique that divides and administers the CM volume over time, previously administered in a single injection. This method has been used to obtain multiple time phases in a single scan [9]. Recently, this technique has been shown to reduce CM usage and yield high-quality images for dual-region assessments, such as coronary and aortic imaging during transcatheter aortic valve replacement [10]. When performing whole-body CT following CTP, imaging is generally performed without adding CM. However, this method makes it difficult to achieve the 200 Hounsfield units (HU) or higher required for diagnosing the pulmonary artery and aorta [9]. To increase CT vessel attenuation values, it is necessary to re-inject CM, which extends examination times. We believe that these problems can be solved by combining SPBI with a low tube voltage.

In this case report, we performed whole-body CT scanning using SPBI and low tube power following brain CTP in a patient suspected of AIS. We report a valuable case in which extremely rare multiple thromboembolisms were diagnosed in a single imaging session.

## Case presentation

A 91-year-old woman was admitted to the hospital with impaired consciousness and left hemiplegia, and a wake-up stroke was suspected. Her last known well time was confirmed to be 1:00 a.m. Initial examination findings included a Glasgow Coma Scale score of E1V3M5, right conjugate gaze deviation, drooping of the left corner of the mouth, and a left upper and lower limb Manual Muscle Test score of 1/5. Her blood pressure was 177/108 mmHg, and her heart rate was 88 bpm (with atrial fibrillation). The patient had previously been independent in activities of daily living and had a history of myocardial infarction (MI) and mitral regurgitation. She had undergone mitral valvuloplasty (MVP) 12 years prior, with uneventful follow-up. CT imaging was performed to assess AIS and determine eligibility for MT.

**Figure 1:**
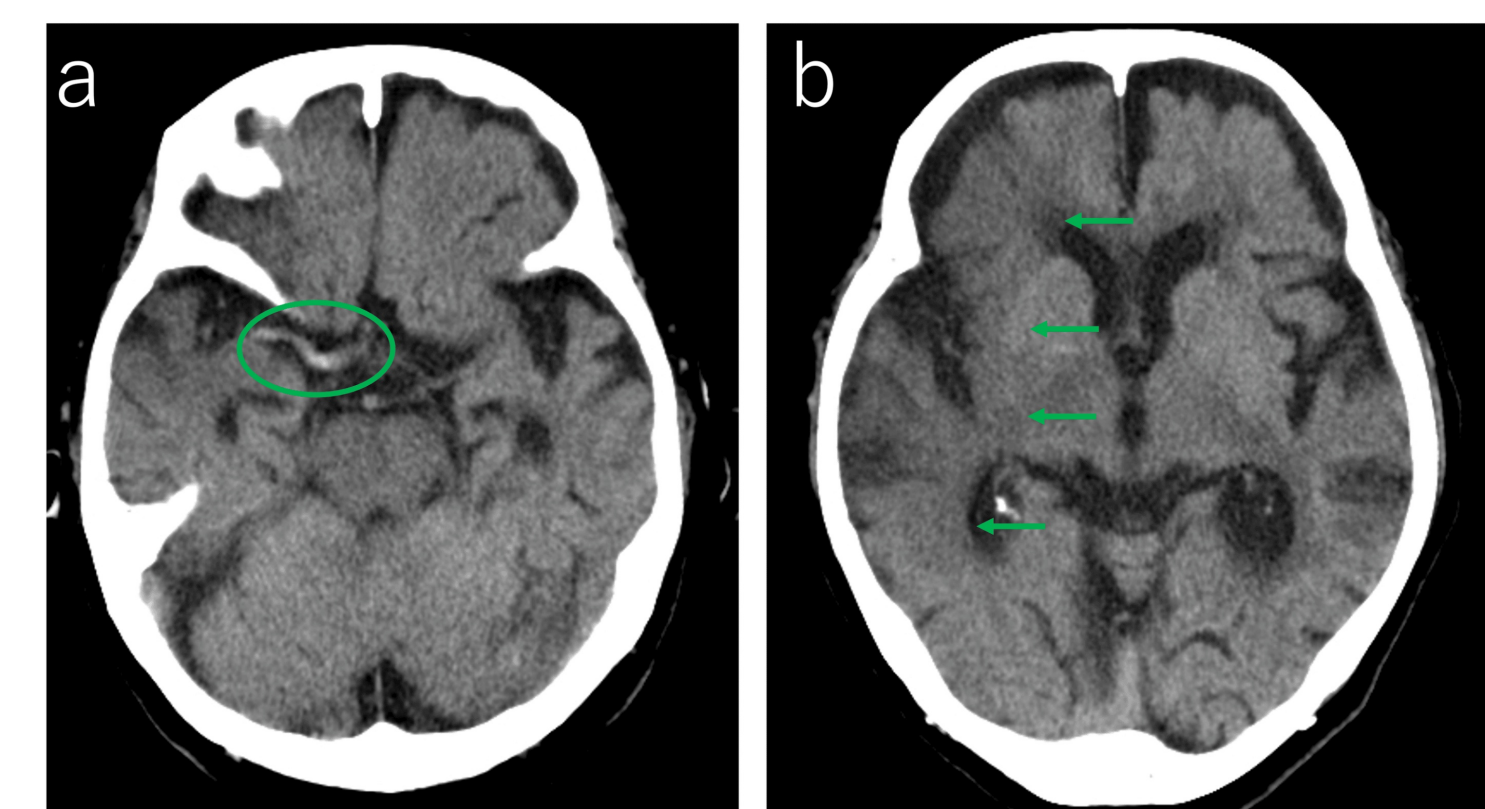
Non-contrast axial brain CT image. (a) A hyperdense sign is observed in the right middle cerebral artery (green circle); (b) loss of gray-white matter differentiation (green arrow). The Alberta Stroke Program Early CT Score (ASPECTS) was rated as 4/10 points.

The CT scanner system used was Revolution CT (GE Healthcare, Milwaukee, WI, USA). Non-contrast brain CT scans revealed a hyperdense sign in the right middle cerebral artery and loss of gray-white matter differentiation in the high fornix. The Alberta Stroke Program Early CT Score (ASPECTS) [11] was 4/10 (Figure 1).

**Figure 2:**
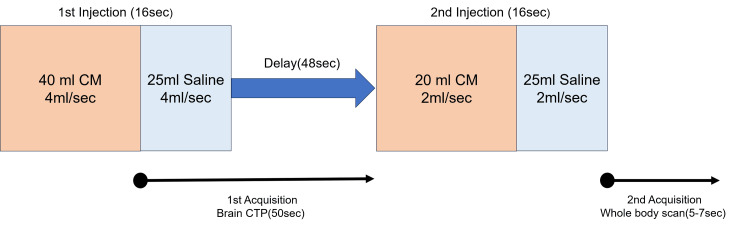
Diagram illustrating the CM injection and imaging procedures for combined brain CTP and whole-body CT using the SPBI method. The protocol involves two CM boluses, separated by a delay, followed by low-tube-voltage scans to enhance vascular visibility while reducing contrast dose. CM: contrast medium; CTP: computed tomography perfusion; SPBI: split-bolus contrast injection Image Credits: Takayuki Inomata

Subsequently, brain and whole-body CT scans were performed. SPBI was used as the CM injection method to optimize vascular enhancement. The laboratory results showed a serum creatinine level of 1.34 mg/dL and an estimated glomerular filtration rate of 29 mL/minute/1.73 m², consistent with severe renal impairment. The total iodine dose was 420 mg/kg. SPBI was administered as follows: in the first phase, 40 mL of CM was injected at 4 mL/second, followed by 25 mL of saline at the same rate. After a 48-second pause, the second phase followed with 20 mL of CM at 2 mL/second, followed by 25 mL of saline at the same rate. Brain CTP was acquired for 50 seconds, starting 10 seconds after the first-phase injection. A whole-body CT scan was acquired immediately after the second injection phase (Figure 2). Imaging parameters were as follows: brain CTP: 80 kVp, 100 mA, 1 second/rotation; whole-body CT: 80 kVp, 100-600 mA (noise index: 12), 0.5 second/rotation, pitch factor: 0.992.

**Figure 3:**
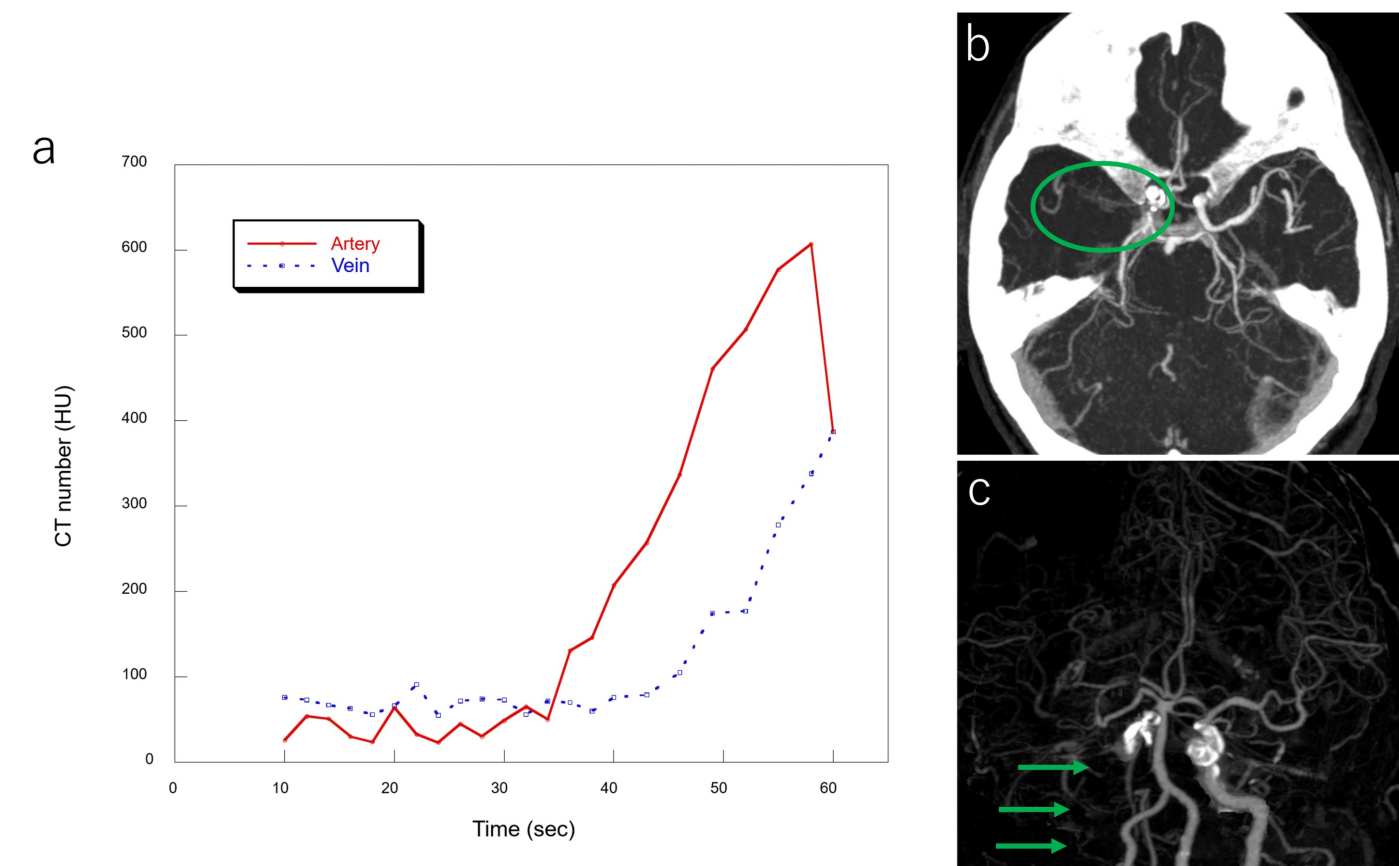
Time–enhancement curve and imaging findings obtained from the brain CTP scan. (a) Time–enhancement curve of CTP. Arterial contrast arrival time was significantly delayed, with the peak enhancement at 58 seconds; venous enhancement did not peak within the scan duration. (b,c) Axial slab-MIP and MIP images derived from CTP data show occlusion of the internal carotid artery (green circle and arrows). CTP: computed tomography perfusion; MIP: maximum intensity projection

In brain CTP, the peak arterial arrival time of CM was delayed to 58 seconds, and the venous peak enhancement was not achieved during the scan period. Therefore, CTP analysis was not possible. The CTP time-enhancement curve is shown in Figure 3a. Axial CT images revealed right internal carotid artery occlusion. Figures 3b, 3c show the maximum intensity projection images.

**Figure 4:**
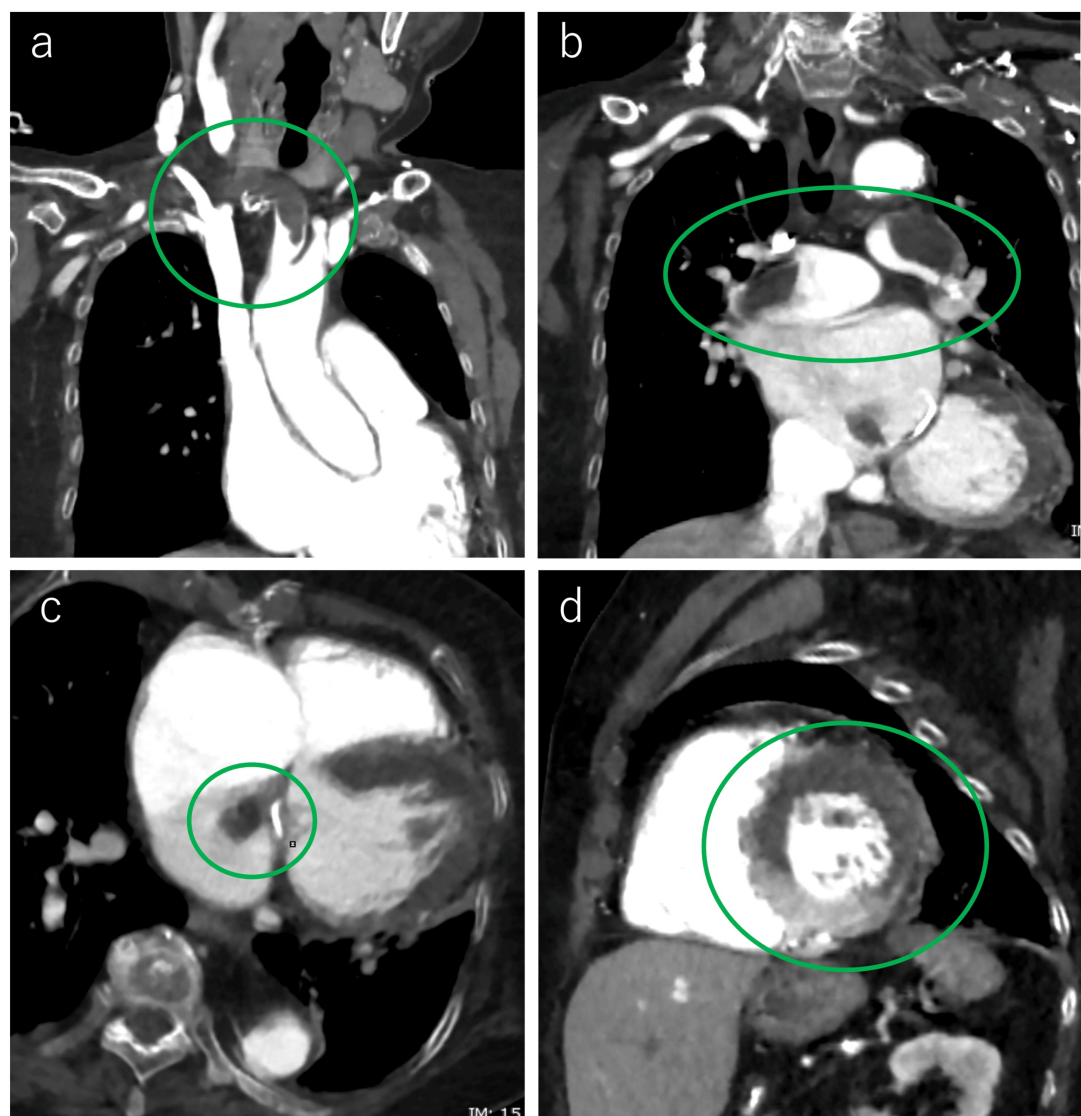
Whole-body CT findings with the lesion indicated by green circles. (a) Coronal image showing thromboembolism at the origin of the brachiocephalic artery, considered the cause of cerebral infarction. (b) Coronal image showing massive thrombi in the main trunks of the left and right pulmonary arteries, indicating pulmonary thromboembolism. (c) Axial image showing a thrombus in the left atrium near the mitral valve. (d) Short-axis cardiac image showing reduced contrast enhancement of the myocardium wall, raising suspicion of myocardial infarction.

Whole-body CT scans revealed multiple large thrombi at the origin of the brachiocephalic artery, in the main pulmonary artery, and adjacent to the mitral valve in the left atrium (Figures 4a-c). Hypoenhancement of the anterior myocardial wall suggested possible MI (Figure 4d).

Subsequent 12-lead electrocardiography showed ST elevation in leads I and aVL and ST depression in leads II, III, and aVF. Laboratory testing revealed a markedly elevated troponin I level (292.00 ng/mL), and the patient was diagnosed with MI. Due to advanced age and the presence of multiple thromboembolic events, invasive therapy was not pursued. Conservative management was selected, but the patient died that evening.

## Discussion

We encountered an exceptionally rare case in which a patient presented with a wake-up stroke and was diagnosed with cerebral infarction (CI) due to thrombotic occlusion at the origin of the brachiocephalic artery, pulmonary embolism (PE), left atrial thrombus, and MI. The simultaneous onset of CI and MI is referred to as cardio-CI [12]. It is extremely rare, with an incidence rate of 0.009% and a very high associated mortality rate [13]. However, the literature on optimal treatment strategies is very limited [10]. In this case, the concurrent occurrence of PE and left atrial thrombus represented an even rarer and more complex condition. The simultaneous occurrence of PE and MI is also considered extremely rare, and debate continues regarding their causal relationship and treatment methods [14]. To our knowledge, there have been no reports of cases in which PE was complicated by cardio-CI. There is no consensus on the management or treatment methods for any of these complications, and further reports are awaited. Although malignant tumors are often associated with the simultaneous onset of PE and arterial thrombosis [15], no malignant tumors were diagnosed in this case. The MI was suspected to have resulted from the rupture of a thrombus in the left atrium. The embolus is believed to have traveled to the brachiocephalic artery, causing occlusion and leading to CI. Furthermore, since atrial fibrillation is a known risk factor for PE, the left atrial thrombus was also considered a likely contributor to the PE [16]. The thrombus was located near the mitral valve, and the possibility of its occurrence due to the effects of previous MVP was also considered. Its rupture likely triggered the cascade of simultaneous thromboembolic events. However, as the MVP had been performed 12 years earlier and no abnormalities had been observed during routine follow-up, this connection could not be confirmed. The patient died on the day of admission, and the underlying cause of the multiple thrombotic events remains undetermined.

In the present case, combined brain CTP and whole-body CT scanning enabled comprehensive diagnosis, including identification of MI and multiple thrombi, in a single imaging session. The CTP revealed a severely delayed arterial peak of 58 seconds, rendering analysis infeasible. We have performed CTP imaging on more than 150 cases using this protocol; this case was the first that could not be analyzed. The time required for the contrast agent to reach the brain varies depending on individual patient factors, such as the injection site, flow rate, and cardiac output. However, a scanning time of 45 to 60 seconds is generally used for CTP [17]. It has been reported that analysis is possible within this time in more than 90% of patients [18]. In this case, imaging was performed for 60 seconds. One method for optimizing imaging time is to first perform a CTA, measure the arrival time, and then adjust the imaging time accordingly [19]. However, this method is not suitable for AIS, where rapid diagnosis is required, due to issues such as the need to add CM and extend the examination time. Further consideration is needed for optimizing CTP imaging time in the future. While such extreme delays are rare, delayed contrast arrival is occasionally observed. In these instances, hemodynamic compromise should be suspected, emphasizing the importance of whole-body imaging and thorough clinical assessment.

By using SPBI, the CM used for CTP imaging can be effectively utilized during whole-body CT imaging, helping achieve high vascular CT attenuation values even with a small amount of CM. Additionally, combining low tube voltage can further enhance CT attenuation values. In this case, we required only an additional 20 mL of CM, yet consistently achieved CT attenuation values of ≥400 HU in major vessels, enhancing diagnostic clarity. It is straightforward to implement, and the extended imaging time is limited to the second CM injection phase (approximately 20 seconds). Therefore, this method is considered highly effective for patients with AIS requiring urgent diagnostic imaging.

The renal effects of intravenous CM are limited, and its appropriate use is generally acceptable [5-7]. However, when MT is indicated in AIS, additional contrast may be required; thus, minimizing contrast volume during CT is essential. This imaging protocol meets AIS diagnostic demands by providing high-quality images while minimizing the amount of CM used.

## Conclusions

This case report highlights the importance of whole-body imaging in patients with AIS, particularly when evaluating for rare but clinically significant multiple thromboembolisms. CT scanning enables the rapid assessment of both the brain and body, providing substantial diagnostic value. Following brain CTP, performing whole-body CT imaging in combination with SPBI and low tube voltage may serve as an effective technique for AIS diagnosis, as it enables high CT attenuation values in large vessels with a simple method and minimal additional CM. This case demonstrates the need for further studies to verify the diagnostic utility of this imaging protocol.
